# Post-Egression Host Tissue Feeding is Another Strategy of Host Regulation by the Koinobiont Wasp, *Toxoneuron nigriceps*

**DOI:** 10.1673/031.011.0103

**Published:** 2011-01-12

**Authors:** Indira Kuriachan, Ruth Henderson, Rachel Laca, S. Bradleigh Vinson

**Affiliations:** Department of Entomology, Texas A & M University, College Station, TX, 77843-2475, USA

**Keywords:** *Heliothis virescens*, host-parasitoid interactions

## Abstract

Koinobiont wasps start their lives as hemolymph feeders inside the host body, but before they egress from the host many become tissue predators. One species, the endoparasitoid *Toxoneuron nigriceps* Viereck (Hymenoptera: Braconidae), exhibits the unusual behavior of egressing before initiating tissue predation. After egression from the host, it reinserts its head into the host body to begin tissue feeding. These third instar *T. nigriceps* larvae show a significant increase in body size and mass after post-egression feeding. Through this project the importance of post-egression feeding in the development of *T. nigriceps* in its host the tobacco budworm, *Heliothis virescens* Fabricius (Lepidoptera: Noctuidae), has been evaluated. The study was conducted by preventing the egressed third instar *T. nigriceps* larvae from feeding on host tissue and observing whether they could undergo further development. Though some of the larvae that were prevented from post-egression feeding did undergo cocoon formation, pupation, and adult emergence they were inferior in terms of size, body mass, and survival to those that developed from larvae allowed to feeding after egression. Hence, it is concluded that post-egression host tissue feeding is essential for the normal development of *T. nigriceps*, as the prevention of feeding resulted in significantly lighter and smaller larvae, cocoons, and adults as well as deformed adults and reduced adult survival. Post-egression feeding as a host regulatory strategy is discussed.

## Introduction

Parasitoids are entomophagous insects that successfully exploit their hosts through specialized mechanisms evolved from the interactions with their hosts (Vinson 1975; Vinson and Iwantsch 1980). The success of parasitoids in locating and exploiting their hosts has made them the most widely used entomophagous insects in classical and applied biological control programs. Parasitoids are free living as adults, but the young of many species are dependent on the adult female selecting a single host from which the immature stages derive all of their nutrition (Vinson et al. 2001). There is no opportunity for the immature parasitoid to choose or move to a different resource. The immature stages of parasitoids are initially parasitic, absorbing nutrients in the host hemolymph, but later in their development they often act as predators (i.e. tissue predators); although they only consume one prey item — the host.

The life histories of parasitic wasps are quite fascinating due to the complexity of their interactions with the hosts. Both idiobionts (whose hosts cease development after parasitism) and koinobionts (whose hosts continue to develop as the parasitoid matures) depend on the resources of a single host to complete their development, and so they have to use different tactics to make use of the maximum available resources for their own development and survival. Most koinobionts are endoparasitoids of larval stage insects, and thus they have more challenges to complete development since they have to compete with the living, growing tissues of the host larva. In order to make the host suitable for the developing parasitoid larva, koinobionts alter or regulate the host physiology for their own benefit. Also, many can and do attack several host stages. For example, *Toxoneuron nigriceps* Viereck (Hymenoptera: Braconidae) can attack first through fifth instar larval hosts, but early instar hosts do not provide the necessary resources. Thus, parasitoids such as *T. nigriceps* delay their development in the host until the host attains the last larval instar (Pennacchio et al. 1993).

The host-parasitoid system of this study is *Heliothis virescens* Fabricius (Lepidoptera: Noctuidae), commonly known as the tobacco budworm, and its endoparasitic koinobiont wasp, *Toxoneuron nigriceps* Viereck (Hymnoptera: Braconidae). *H. virescens* is an economically important pest that attacks cotton, tobacco, and various vegetable and flower crops. The larval stages of *H. virescens* usually attack the unfolded leaves, flower buds, and ovaries of developing flowers that reduces the quantity and quality of these crops (Huffaker 1985; Metcalf and Metcalf 1993). Considering the environmental problems associated with the use of insecticides and the insecticide resistance exhibited by the pest insects, biological control has become one of the important components of integrated pest management programs. *Toxoneuron nigriceps*, formerly known as *Cardiochiles nigriceps* (Whitfield and Dangerfield 1997), is an effective biological control agent of *H. virescens*. Once the wasp oviposits in the *H. virescens* larva, the mortality of the caterpillar is highly likely. Further, *T. nigriceps* has served as a model for several important discoveries in parasitoid biology, such as the discovery of and studies related to polyDNA viruses (Vinson and Scott 1974c; Stoltz and Vinson 1979; Tanaka and Vinson 1991), role of teratocytes (Vinson and Scott 1974b; Pennacchio et al. 1991, 1994c), evolving host immune mechanisms (Lewis and Vinson 1968; Vinson 1971, 1990, 1993), host location (Vinson and Lewis 1965; Vinson 1968; Hays and Vinson 1971), and parasitoid learning (Vinson 1976, 1991).

Another important advancement made with *T. nigriceps* has been the success achieved in its *in vitro* rearing, especially as koinobionts have remained the most challenging group of parasitoids to rear on an artificial medium. Previously, *T. nigriceps* has been reared by Pennacchio et al. (1992) from post-germ band egg to second instar larva in an artificial diet devoid of any insect material. Consoli and Vinson (2004) improved the *in vitro* development of 6 h-old eggs of *T. nigriceps* by adding host factors released by the host fat body to the artificial medium. Based on the information on host hemolymph proteins and parasitoid development an artificial diet supplemented with host hemolymph has been successfully developed for early second instar *T. nigriceps*, in which 100% of the larvae molted to the third instar (Kuriachan et al. 2006). However, all of the third instar larvae appeared to be transparent and fragile compared to the white and sturdy appearance of those reared *in vivo*. The third instar larvae reared *in vitro* demonstrated behavioral changes, similar to those of newly egressed third instar *T. nigriceps* larvae *in vivo*, that could be interpreted as the preparation for reinserting the head into the host or cocoon formation (i.e. oral secretion of a whitish material as well as twisting and turning movements); however, none developed further or produced a cocoon or pupa. This prompted the hypothesis that there are some key nutrients that are lacking in the artificial diet for the final *in vitro* development of the third instar larvae.

**Figure 1.**
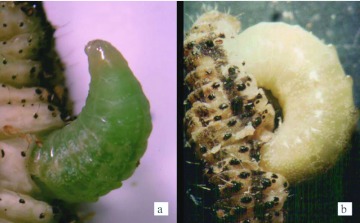
a) *T. nigriceps* larva egressing with head first through the second abdominal segment of the host. The egressing larva is light green in color, b) Egressing *T. nigriceps* larva reinserted its head into the posterior end of the host between the fifth and sixth abdominal segments of the host. The larva became plump, opaque, and whitish in color. High quality figures are available online.

On reaching the third larval instar *in vivo*, the *T. nigriceps* larva that is light green in color, egresses from the host (Figure 1a). Egression occurs by making a hole in the host's cuticle near the second abdominal segment on the side, big enough to first push its head out and then slowly push the anterior part of the body out of the host. As soon as the head and ¾ of the body is out of the host, the larva curls, makes a hole in the host's cuticle, and reinserts the head into the host posterior to its emergence hole between the fifth and sixth abdominal segment and begins to feed on the host tissue as shown in Figure 1b (a larva that has been feeding). While feeding, the anterior portion of the host is moved closer to the feeding larva as the host tissue is consumed. After host tissue feeding, the egressed larva shows a significant increase in size and, presumably, body mass as well as a change in color. These observations on the final stage of the third instar larvae *in vivo* and *in vitro* stimulated interest in investigating the importance of host tissue feeding on the final development of *T. nigriceps*. The objective of this study was to determine the effects of post-egression host tissue feeding on *T. nigriceps* development. This was achieved by 1) observing the development of *T. nigriceps* larvae without post-egression feeding, 2) comparing the body mass of the larvae, cocoons, and adult wasps that were or were not allowed to feed on host tissue following egression from the host, and 3) determining the percentage of larvae undergoing cocoon formation, pupation, and adulthood as well as survival of the adults.

## Materials and Methods

### Insect cultures and rearing

The host, *H. virescens*, was reared on an artificial diet (Tobacco budworm diet, BioServ, Inc., www.bio-serv.com) (Vanderzant et al. 1962) under controlled conditions (29±1° C; 60±10% RH; 14:10 L:D). The parasitoid, *T. nigriceps*, was reared as described by Vinson et al. (1973).

The larval stages of the host, *H. virescens*, were identified following the morphological descriptions by Webb and Dahlman (1985). Fourth instar larvae at the head capsule slippage stage were used for parasitization. The larvae were individually parasitized by placing one *H. virescens* larva and a mated *T. nigriceps* female together in a parasitization chamber (30 mm × 5 mm petri dish). The parasitized larvae were immediately transferred to the rearing container (6 mm × 2 mm plastic vials containing a 12 mm × 12 mm piece of the artificial diet) under controlled conditions (29±1° C; 60±10% RH; 14:10 L:D). In order to synchronize the larval development, only larvae that molted to fifth instar within the first 12 h after parasitization were used for the study. Parasitized larvae were removed from the diet to empty test tubes on the 11^th^ day after parasitization. Normally, third instar *T. nigriceps* larvae egress out from the host on the 11^th^ or 12^th^ day after parasitization (personal observation). These larvae were closely observed for the beginning of parasitoid egression.

The experimental larvae consisted of two groups. The larvae in the first group were allowed to feed on host tissue as they normally do, a process that usually requires 2– 3 hours. The larvae in the second group were prevented from post-egression feeding by gently moving the host's body away from the egressing *T. nigriceps* larva's head, thus obstructing the reinsertion of its head into the host's body for 3 hours. After 3 hours, the larvae from both groups were weighed and placed individually in 0.5ml and 6.63mm gel capsules (Electron Microscopy Sciences, www.emsdiasum.com) that served as artificial pupation chambers that were found to be suitable for successful cocoon formation and pupation (Henderson et al. in preparation). Cocoon formation and adult emergence were observed, and the masses of the egressed larvae, cocoons, and adults were recorded. Adult longevity was also recorded.

Twenty-five *T. nigriceps* larvae in each group (larvae with and without post-egression feeding) were used to determine the effect of post-egression feeding on the mass of third instar larvae, cocoons, and adults. Fifty larvae were used in each group to compare the number of larvae formed with no cocoons, the number of adults that emerged from cocoons in each group, and the number of adults in each group that survived more than one week after emergence.

## Data analysis

Statistical analyses were performed using JMP® Statistical Discovery Software, Version 4 (2001 SAS Institute Inc. version 7, www.sas.com). A two-tailed student t-test was used to compare the mass of the larvae, cocoons, and adults between groups with and without post-egression feeding. A χ^2^ test was used to compare the number of cocoons formed, adults emerged, and adults that survived up to one week after emergence.

## Results

### Larval Mass

**Figure 2.**
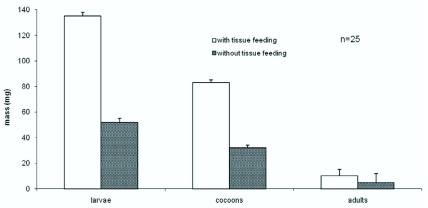
Body mass of larvae, cocoons, and adults in treatments with and without host tissue feeding after egression from the host. Larval mass, cocoon mass, and adult mass were significantly different (t-test, p<0.000l) between the two groups (with and without tissue feeding after egression). Data are shown as means ± SE. High quality figures are available online.

There was a significant difference in larval mass between third instar *T. nigriceps* larvae that were allowed to feed after egression and those that were prevented from feeding (t= 19.07, df = 48; p <0.0001) (Figure 2). The larvae allowed to tissue feed had greater mass and were plump, opaque, and whitish in color due to the enlarged fat body cells, which could be seen through the thin and clear cuticle as shown in Figure 1b. This was in contrast to the larvae with no tissue feeding after egression, which remained small and appeared light green as in Figure 1a due to the hemolymph inside the larva.

### Cocoon Mass

**Figure 3.**
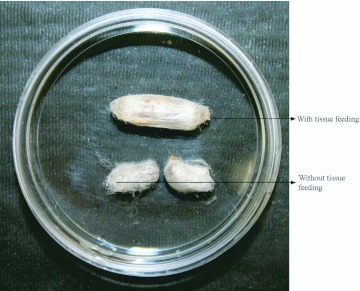
Comparison of the size of the cocoons from treatments with and without tissue feeding after egression. The cocoons formed from the larvae without post-egression tissue feeding were less than half the size of the cocoons formed from the larvae with post-egression tissue feeding. High quality figures are available online.

As demonstrated in Figure 2, cocoons formed by larvae with post-egression feeding and those without also exhibited a significant difference in mass (t = -16.61; df = 41; p <0.0001). Cocoons that developed from the larvae without post-egression feeding were approximately half the size of those that developed from larvae allowed to feed on host tissue (Figure 3).

### Adult Mass

The adult wasps that emerged from larvae without post-egression feeding had significantly smaller mass than those that emerged with host tissue feeding (t = -7.03; df = 35; p < 0.0001) (Figure 2).

### Cocoon formation

**Figure 4.**
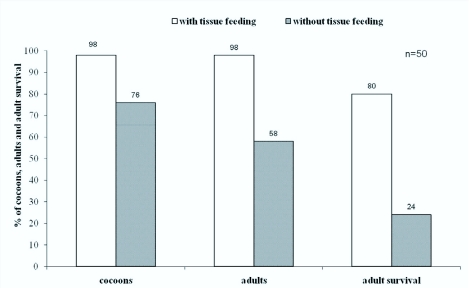
Percentage of cocoons formed, adults emerged and adults that survived for I week in treatments with and without post-egression host tissue feeding. There were significant differences (χ^2^ test, p<0.0001) in number of cocoons formed, adults emerged and adults survived between the two groups (with and without tissue feeding after egression). High quality figures are available online.

In the treatment where larvae were allowed to feed after egression 98% of the larvae formed cocoons, while in the treatment in which larvae were prevented from tissue feeding only 76% were able to form cocoons (Figure 4). The rest of the larvae either died or did not succeed in their effort to form cocoons. There was a significant difference in cocoon formation between the two treatments (χ^2^ =12.37; df = 1; p < 0.0004). Eighteen percent of the larvae that were prevented from tissue feeding did not form cocoons, but did pupate; however, they were unable to excrete the meconium and failed to develop further. The remaining 6% died as third instar larvae without undergoing any further development.

### Adult emergence

There was significant difference in the number of emerged adults between the two treatments (χ^2^ = 42.03; df = 1; and p < 0.0001). In the treatment where larvae were allowed to feed on tissue a normal adult wasp emerged from every cocoon, whereas in the treatment where larvae were prevented from tissue feeding only 58% developed into normal adults (Figure 4). However, these were half the size of the adults of their counterparts. There were 28% deformed adults and 14% found dead inside the cocoons. In the deformed, living adults 70% lacked complete wing development and 30% had only wing pads.

### Adult Survival

In the treatment with host tissue feeding, 80% of the adult wasps that developed were alive for more than 4 weeks as normally occurs under laboratory conditions. Only 20% of the emerged adults in treatment with restricted host tissue feeding survived for 1 week (Figure 4). There was a significant difference between these two treatments (χ^2^ = 47.73; df = 1; and p < 0.0001).

## Discussion

Prevention of post-egression tissue feeding produced significantly lighter and smaller larvae, cocoons, and adults as well as deformed adults and reduced adult survival. The percentage of larvae that were able to reach adulthood was also less than that of larvae allowed to feed after egression. Providing a suitable pupation chamber (gel capsules) resulted in successful cocoon formation in both treatments; however, many of the adults in treatment with restricted postegression tissue feeding failed to emerge from their cocoons. Others had some developmental abnormalities, such as lacking complete wings. This might be due to a deficiency in energy, nutrients, vitamins, and/or minerals. In addition, there was a positive impact on the longevity of the adults with post-egression tissue feeding. Though the adult wasps emerged without post-egression tissue feeding were alive for a few days (less than one week) in the laboratory under controlled conditions, in the field survival may not be possible due to weather and natural enemies. Thus the study leads to the conclusion that post-egression tissue feeding is mandatory for the normal development and survival of *T. nigriceps*.

Body size is one of the most important life history characters of an organism and its effects on fitness have been well documented (Calder 1984; Schmidt-Nielsen 1984; Roff 1992; Stearns 1992). Being small may facilitate a safe egression. Post-egression tissue feeding may allow the parasitoid larva to attain a critical mass that is necessary for its adult emergence and survival. In order to emerge from the host, some basal pressure (from terminal body segments) needs to be applied by the wasp larvae from within the host (Nakamatsu et al. 2007). Reinsertion of the head into the host body may serve as an anchor for the egression of the rest of the parasitoid's body; thus reducing the energy utilization. As mentioned previously, after reinserting its head the egressing parasitoid curls up with both ends inside the host body, thus having the anterior end as the anchor while pushing the posterior end outside (Figure 1b).

Parasitoids disrupt normal biochemical (Thompson 1983), nutritional (Vinson et al. 2001), physiological (Beckage 1985), and behavioral (Brodeur and McNeil 1989) patterns of their host for their own benefit. While inside the host's hemocoel, koinobionts change or regulate the hormone titers and other proteins of the host via teratocytes, PolyDNA viruses, calyx fluid, etc. (Beckage et al. 1994; Wani et al. 1990, Pennacchio et al. 1994b). Even upon egression, the parasitoids exploit the host in different ways. Another braconid parasitoid, *Microplitis croceipes*, also parasitizes *H. virescens*. However, *M. croceipes* exploits the host in a different way after egression. Unlike *T. nigriceps*, which starts consumption of the host tissue and kills the host while egressing, *M. croceipes* egresses from the host without causing much damage to the host body leaving the host alive for a few days after parasitoid egression. In this case, the parasitoid usurps the behavior of the living host so that the caterpillar guards the egressed parasitoid larva and the resulting cocoon. The host larva coils on the parasitoid cocoon to protect or hide it from predators. Usurpation behavior is reported in some other cases as well. A gregarious braconid wasp, *Cotesia glomerata*, causes its moribund host caterpillar, *Pieris brassicae*, to remain on the pupating parasitoids, spin a web over the parasitoid cocoons, and to respond aggressively when disturbed (Brodeur and Vet 1994; Harvey et al. 2008). Both *M. croceipes* and *C. glomerata* pupate on the surface of the leaves, which is unsafe for the parasitoid's survival, so they manipulate the host's behavior to safeguard the cocoons. After egression *T. nigriceps* larvae are protected within a host-formed pupation chamber beneath the soil, so they do not need any more protection from the host. However, they do need a strong cocoon to protect the pupa sealed underground. To make a strong cocoon, the egressed larva should produce a significant quantity of silk. Post-egression feeding may provide the energy and protein needed for cocoon formation. *T. nigriceps* is also a much larger wasp than *M. cropceipes* and *C. glomerata*, and thus it likely needs more nutrients for normal development. By consuming all host tissues after egression, *T. nigriceps* obtains nutrients essential for further development. This may be another form of host regulation for the parasitoid's own benefit.
